# Neuroblastoma Soft Tissue Metastasis in a 10-Month-Old Infant with a Right Thigh Mass

**DOI:** 10.1155/2021/3226319

**Published:** 2021-09-01

**Authors:** Shokouh Taghipour Zahir, Fateme Salemi

**Affiliations:** ^1^Department of Pathology, Shahid Sadoughi General Hospital, Yazd, Iran; ^2^Islamic Azad University of Medical Sciences, School of Medicine, Yazd, Iran

## Abstract

**Background:**

Neuroblastoma is a solid tumor that occurs more frequently in pediatric populations. It may originate from any part of the sympathetic nervous system, but it most commonly arises from the paraspinal sympathetic ganglia in the abdomen or mediastinum. Local lymphadenopathy and distant metastasis to the central nervous system, orbit, and liver might be detected; however, it rarely includes soft tissue or musculoskeletal involvement. *Case Report*. Here, we report a 10-month-old infant presented with a right thigh mass with an otherwise benign physical exam and medical history. MRI of the lower extremities suggested tumoral infiltration in the soft tissue of both thighs, predominantly on the right side. Surgical pathology of the lesion confirmed neuroblastoma. A large subhepatic mass and paraaortic lymphadenopathy in the abdominal CT scan and metaiodobenzylguanidine scan findings favored primary abdominal neuroblastoma that had spread to lower extremities. The patient has been in remission since the completion of chemotherapy.

**Conclusion:**

Neuroblastoma should be considered in infants with an abnormal mass in extremities. Due to its aggressive nature, most patients struggle with distant and local tumor spread at diagnosis. Therefore, any abnormal signs and symptoms, especially in younger pediatrics, warrant immediate evaluation to avoid tumor expansion.

## 1. Introduction

Neuroblastoma originates from neural crest cells of the sympathetic nervous system. More specifically, it develops from the paraspinal sympathetic ganglia in the abdomen or mediastinum. Neural crest cells migrate to form the adrenal medulla and paraspinal mediastinal or abdominal sympathetic ganglia [1]. As the third most common pediatric solid tumor, neuroblastoma comprises 6–10% of all childhood cancers and 15% of mortalities [2, 3]. It has a higher incidence in males and Caucasians [4]. In addition to the existence of sporadic neuroblastoma, there is also a rare hereditary type associated with both Hirschsprung disease and neurofibromatosis type 1 [5]. Patients may present with fever, weight loss, malaise, dyspnea, dysphagia, lethargy, anorexia, irritability, and/or pale skin, and the last is reported in about 33% of patients [6, 7]. Orbital and cranial infiltration of neoplastic cells often manifests as bone pain [8]. Systemic symptoms such as fever, anorexia, watery diarrhea, and hypertension may result from acute phase reactants and catecholamines release [6]. Almost all patients showed elevated urinary catecholamines, such as homovanillic acid (HVA) and vanillylmandelic acid (VMA) [9]. Histopathology demonstrates small, round, blue tumoral cells in varying stages of differentiation, making immunohistochemistry (IHC) staining necessary to confirm the diagnosis [10].

Like other malignancies, staging and risk stratification are essential to design an appropriate treatment plan [3, 11]. As in this case, low and intermediate-risk patients respond to chemotherapy alone; however, high-risk neuroblastomas require a multidisciplinary approach. In this report, a rare case of neuroblastoma with bone and skeletal muscle metastases is presented. We emphasize that any abnormal mass, especially in children younger than two years old, should raise suspicion for this solid tumor.

## 2. Case Presentation

**Figure 1:**
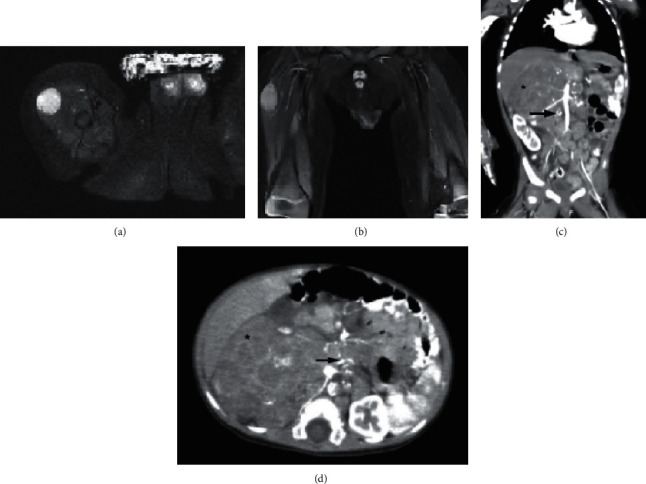
Initial axial T2-weighted MRI revealed the soft tissue lesion with a hyperintense signal in axial (a) and coronal (b) views. Abdominopelvic CT scan coronal (c) and axial (d) views show a heterogenic mass (black stars) in the subhepatic along with paraaortic calcified lymph nodes (black arrows).

**Figure 2:**
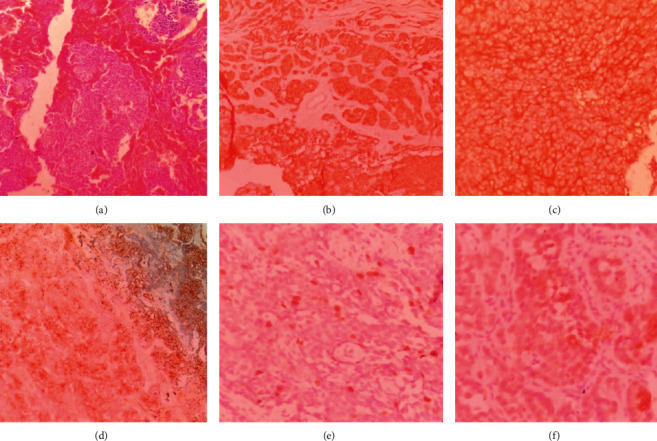
Hematoxylin and eosin (H&E) staining showing round cells with scanty cytoplasm arranged in solid nests with pseudo rosette formation (a). In IHC staining, neoplastic cells demonstrate positive immunoreactivity for NSE (b), CD56 (c), and vimentin (d) and negative immunoreactivity for synaptophysin (e) and cytokeratin AE1/AE3 (f).

In December 2019, a 10-month-old infant with no significant medical history presented with a mass in the anterior aspect of the right thigh found by his parents three months prior. No other symptoms were reported. Magnetic resonance imaging (MRI) of both thighs without contrast with axial, sagittal, coronal T1W, PD, and PDFS sequences showed several fusiform intramuscular and intermuscular lesions up to 15 × 14 mm in size at both thighs, more prominent on the right side, suggestive of neurofibroma. The bone and bone marrow each appeared normal and devoid of any lytic or sclerotic lesion (Figures 1(a) and 1(b)). One week later, the patient underwent surgery for radical excision of the thigh mass with clear margins. In gross pathology, a grayish tumor 3 × 2 cm in size with hemorrhagic areas was inspected. Histopathology demonstrated the accumulation of small, dark, round cells with scant cytoplasm arranged in solid nests with pseudo rosette formations along with areas of hemorrhage (Figure 2(a)). Immunohistochemistry showed positive immunoreactivity for neuron-specific enolase (NSE), CD56, vimentin, and chromogranin A. Lack of staining for synaptophysin and cytokeratin AE1/AE3 confirmed the diagnosis of neuroblastoma (Figures 2(b)–2(f)).

During a follow-up appointment, a right upper quadrant abdominal mass was noted. Abdominopelvic ultrasound displayed an ill-defined hypoechoic lesion measuring 115 × 68 × 67 mm with calcification and vascularity. It was located between the liver and the right kidney and seemed to cross the midline, highly suggestive of malignancy. Subsequently, an abdominopelvic CT scan with contrast demonstrated a large heterogeneous mass in 74 × 70 mm in the subhepatic area with paraaortic and pelvic calcification centers suggesting lymphadenopathy (Figures 1(c) and 1(d)).

To identify any undetected neoplastic lesion, the patient underwent Metaiodobenzylguanidine (MIBG) scan, which showed the increased uptake of the primary soft tissue mass in the subhepatic region. In contrast to MRI findings, it revealed metastasis to the superior part of the right femur and its adjacent soft tissue. Our patient was classified as intermediate-risk despite having distant metastases because the fluorescence in situ hybridization (FISH) test was negative for MYCN gene amplification. The abdominal tumor was managed without surgical removal, and the patient only received VAC chemotherapy regimen (vincristine, actinomycin-D, and cyclophosphamide). After two years, he has been doing well and has not shown any signs of tumor relapse.

## 3. Discussion

As a high-grade solid malignancy, the clinical presentation of neuroblastoma depends on the location of the primary and metastatic lesions [8]. This neoplasm often stems from the abdomen and locally invades adjacent lymph nodes [12]. It may spread via the bloodstream to the bone marrow, bone, liver, soft tissue, orbital and intracranial structures, and skin; however, the lung is rarely involved through lymphatic tumor spread [13, 14]. Therefore, patients might present with abdominal mass, bone pain, and preorbital ecchymosis commonly referred to as “raccoon eyes” [8]. Bone marrow engagement elicits significant pain and increases the risk of fracture and pancytopenia [14]; however, our case was asymptomatic aside from the right thigh mass. Skull involvement is detected in nearly one-third of the patients. Bone and soft tissue metastasis happen in only 15% of patients diagnosed with neuroblastoma [15]. Özger et al. reported an ill and febrile 11-month-old girl presented with a warm, painful knee appearing to be a pathological fracture from neuroblastoma bone involvement [16]. In another study, a neonate with a history of intrauterine growth restriction was diagnosed as neuroblastoma with metastasis to cardiac and skeletal muscles. MRI demonstrated subcutaneous nodules and bone marrow involvement with similar signals highly suggestive of metastasis; however, MRI, in this case, suggested a benign lesion [17]. However, to the best of our knowledge, this is the first reported case of neuroblastoma with muscular involvement and intact bone marrow. CT scan or MRI following abdominal ultrasound can detect the primary tumor location, and MIBG scintigraphy (a norepinephrine analog) with I-123 is the gold standard modality to detect distant metastasis [18].

In histopathological assessments, primary or metastatic lesions appear as small round blue cells that require IHC staining to confirm the diagnosis. Homer Wright pseudorosettes, an eosinophilic neuritic process (neuropil) of neuroblasts, might be seen in some cases [19]. IHC staining shows positive immunoreactivity for specific enolase, chromogranin A, neurofilament protein, S100, and synaptophysin, differentiating it from other small round cell tumors such as non-Hodgkin's lymphoma, Ewing's sarcoma, rhabdomyosarcoma, and Wilms tumor. However, leukocyte common antigen, vimentin, myosin, desmin, and actin markers do not stain in neuroblastoma [20].

The primary tumor and metastatic lesions of neuroblastoma may present as specific signs and symptoms that can be mistaken for another malignancy. In patients with an abdominal mass, Wilms tumor, hepatoblastoma, and rhabdomyosarcoma must be ruled out [21]. The most frequent pediatric abdominal neoplasm, Wilms tumor or nephroblastoma, shares some clinical findings with neuroblastoma such as the presence of an abdominal mass, discomfort, fever, and elevated blood pressure. Ultrasound will differentiate the Wilms tumor as a renal mass [22]. Additionally, rhabdomyosarcoma is the most common soft tissue sarcoma in pediatric population. Muscles and soft tissues of the extremities or abdominopelvic area are often involved in this malignancy. It must also be differentiated from neuroblastoma through paraclinical evaluations and genetic and imaging studies [23]. Thoracic tumors such as lymphoma and germ cell tumors will be present in the anterior mediastinum, while thoracic neuroblastoma is located in the posterior mediastinum near the sympathetic chain [24].

Bone metastasis in neuroblastoma should be distinguished from primary bone tumors including Ewing sarcoma, osteosarcoma, and chondrosarcoma through imaging features. Ewing sarcoma, a neuroectodermal derivative consisting of poorly differentiated small round or spindle cells, is seen in adults and children older than five. It often arises from the pelvic area, chest wall, and tissues surrounding the spinal cord and bones of the extremities. Systemic symptoms, including fever, weight loss, and elevated erythrocyte sedimentation rate (ESR), are likely in both malignancies [25]. Despite clinical and histologic similarities, ganglioneuroblastoma cells will be more differentiated than neuroblastoma [26]. In this specific case, retinoblastoma was not included in the differential given the normal ophthalmologic exam [27]. Bone marrow involvement in pediatric lymphoma and leukemia can be differentiated from neuroblastoma clinically as constitutional symptoms and lymphadenopathy are more prominent in those cancers [28].

Factors that impact the prognosis and outcome of neuroblastoma, including histopathologic differentiation, tumor stage, age, and MYCN mutation status, categorize patients into low, intermediate, and high-risk groups [29]. Despite various staging systems for neuroblastoma classification, the International Neuroblastoma Staging System (INSS) and International Neuroblastoma Risk Group Staging System (INRGSS) are commonly used in practice. The latest system, INRGSS, has evolved from the INSS, which adds imaging results to clinicopathologic features of patients [3, 30]. Although our patient presented with soft tissue metastasis at the disease onset, negative MYCN mutation and his young age improved his overall outcome. Therefore, his cancer was classified as stage III according to the INSS within the intermediate-risk group [11]. While advanced stages carry an unfavorable prognosis and require multidisciplinary approach, most intermediate-risk patients such as this case reach the disease-free survival period with chemotherapy.

Neuroblastoma often originates from the abdominal organs and rarely spreads to musculoskeletal tissues. Any abnormal mass in a child, significantly less than two years old, warrants thorough clinical and paraclinical evaluations, and the differential should include neuroblastoma.

## Data Availability

The case report data used to support the findings of this study were supplied by Fateme Salemi under license and so cannot be made freely available. Requests for access to these data should be made to Fateme Salemi (fatemesalemi77@gmail.com).

## Abbreviations

MRI: Magnetic resonance imaging
CT: Computed tomography
MIBG: Metaiodobenzylguanidine
HVA: Homovanillic acid
VMA: Vanillylmandelic acid
IHC: Immunohistochemistry
NSE: Neuron-specific enolase
FISH: Fluorescence in situ hybridization
INSS: International Neuroblastoma Staging System
INRGSS: International Neuroblastoma Risk Group Staging System.
